# Exploring the dynamic three-dimensional chromatin architecture and transcriptional landscape in goose liver tissues underlying metabolic adaptations induced by a high-fat diet

**DOI:** 10.1186/s40104-024-01016-5

**Published:** 2024-05-02

**Authors:** Guangliang Gao, Rui Liu, Silu Hu, Mengnan He, Jiaman Zhang, Dengfeng Gao, Jing Li, Jiwei Hu, Jiwen Wang, Qigui Wang, Mingzhou Li, Long Jin

**Affiliations:** 1 Livestock and Poultry Multi-Omics Key Laboratory of Ministry of Agriculture and Rural Affairs, College of Animal Science and Technology, Sichuan Agricultural University, Chengdu, 611130 China; 2 Chongqing Engineering Research Center of Goose Genetic Improvement, Institute of Poultry Science, Chongqing Academy of Animal Sciences, Rongchang District, Chongqing, 402460 China

**Keywords:** Compartment A/B, Goose fatty liver, Promoter-enhancer interactions, Regulation of gene expression, Three-dimensional chromatin architectures, Tolerance hepatic steatosis, Topological domains

## Abstract

**Background:**

Goose, descendants of migratory ancestors, have undergone extensive selective breeding, resulting in their remarkable ability to accumulate fat in the liver and exhibit a high tolerance for significant energy intake. As a result, goose offers an excellent model for studying obesity, metabolic disorders, and liver diseases in mammals. Although the impact of the three-dimensional arrangement of chromatin within the cell nucleus on gene expression and transcriptional regulation is widely acknowledged, the precise functions of chromatin architecture reorganization during fat deposition in goose liver tissues still need to be fully comprehended.

**Results:**

In this study, geese exhibited more pronounced changes in the liver index and triglyceride (TG) content following the consumption of the high-fat diet (HFD) than mice without significant signs of inflammation. Additionally, we performed comprehensive analyses on 10 goose liver tissues (5 HFD, 5 normal), including generating high-resolution maps of chromatin architecture, conducting whole-genome gene expression profiling, and identifying H3K27ac peaks in the livers of geese and mice subjected to the HFD. Our results unveiled a multiscale restructuring of chromatin architecture, encompassing Compartment A/B, topologically associated domains, and interactions between promoters and enhancers. The dynamism of the three-dimensional genome architecture, prompted by the HFD, assumed a pivotal role in the transcriptional regulation of crucial genes. Furthermore, we identified genes that regulate chromatin conformation changes, contributing to the metabolic adaptation process of lipid deposition and hepatic fat changes in geese in response to excessive energy intake. Moreover, we conducted a cross-species analysis comparing geese and mice exposed to the HFD, revealing unique characteristics specific to the goose liver compared to a mouse. These chromatin conformation changes help elucidate the observed characteristics of fat deposition and hepatic fat regulation in geese under conditions of excessive energy intake.

**Conclusions:**

We examined the dynamic modifications in three-dimensional chromatin architecture and gene expression induced by an HFD in goose liver tissues. We conducted a cross-species analysis comparing that of mice. Our results contribute significant insights into the chromatin architecture of goose liver tissues, offering a novel perspective for investigating mammal liver diseases.

**Supplementary Information:**

The online version contains supplementary material available at 10.1186/s40104-024-01016-5.

## Background

High-fat diet (HFD) disrupt lipid metabolism in humans and mammals, leading to liver conditions such as fatty liver, hepatic steatosis, cirrhosis, and liver cancer [1]. Conversely, geese demonstrate an extraordinary physiological capacity to withstand significant fat accumulation caused by HFD without experiencing the usual inflammatory and fibrotic reactions observed in humans and mammals [2–6]. Notably, this capacity is reversible; halting the HFD enables the liver of a goose to revert to its original condition, illustrating its remarkable ability to resist the liver damage typically induced by high-fat consumption [7]. These characteristics highlight the distinct capacity for lipid deposition and hepatic steatosis tolerance observed in goose livers. During short-term HFD (approximately two to three weeks), lipid deposition exceeds β-oxidation, resulting in goose fatty liver formation and increased lipid accumulation [4, 8, 9].

Integrating multiple omics datasets provides a comprehensive approach to investigating mechanisms underlying lipid protection in goose liver. By utilizing the goose genome sequence, transcriptomics, epigenomics, proteomics, gut microbiota metagenomics, and metabolomics, we deepened our understanding of the intricate interactions among different omics levels, contributing to our comprehension of lipid metabolism regulation in geese [4, 9–12]. Studies on the goose genome sequence revealed positive selection-induced deletion of the goose leptin gene, triggering the development of liver mechanisms for energy storage and tolerance to severe hepatic steatosis [4]. Recent investigations using transcriptomics, proteomics, and gut microbiota metagenomics identified numerous genes involved in fat synthesis, lipoprotein transport, fatty acid oxidation, endoplasmic reticulum stress, insulin resistance, hepatocyte growth, and proliferation [9, 13]. Previous studies have elucidated the protective effects of adiponectin and its receptors, as well as components such as Complement 3 (*C3*), Complement 4 (*C4*), Complement 5 (*C5*), and intestinal microbes, on goose livers exposed to HFD [9]. Additionally, our laboratory utilized RNA sequencing data (RNA-Seq) to analyze essential candidate genes, including stearoyl-coenzyme A desaturase (*SCD*), fatty acid desaturase 1 (*FADS1*), and apolipoprotein B (*APOB*), studying the dynamic response to high energy intake [14].

The high-throughput chromosome conformation capture (Hi-C) technology is potent for examining gene expression regulatory mechanisms by exploring spatial interactions among chromosomes across genomes [15]. It allows for examining various structural aspects of chromosomes [16]. These structural features (Compartments, topologically associated domains (TADs), or promoter–enhancer interactions (PEIs)) emphasize the crucial role of three-dimensional changes in gene expression. Hi-C has been used to aid in genome assembly, construct whole-genome haplotypes in mammals, compare chromatin interactions between different cells or species, explore resulting differences in gene expression, and investigate developmental patterns and mechanisms of complex diseases [17]. For example, previous studies have successfully generated three-dimensional chromatin structure maps for porcine cells, enabling real-time tracking of chromatin spatial structure reprogramming during early embryonic development [18]. Investigations have also focused on the early developmental processes of the porcine liver [19].

Additionally, researchers have leveraged the latest porcine reference genome and performed de novo assembly of genomes from 11 geographically and phenotypically diverse pig breeds worldwide, establishing a comprehensive pan-genome for pigs, enhancing our understanding of genetic variations among breeds and facilitating in-depth exploration of the molecular mechanisms that govern transcriptional regulation of pork quality traits [20]. Moreover, Hi-C has been instrumental in reconstructing porcine adipose tissue's three-dimensional genomic spatial structure, providing fundamental data and theoretical support for advancements in molecular genetic breeding [21]. Similar research endeavors have been conducted in various livestock species, including chickens [22], ducks [23], and model organisms such as mice [24] and zebrafish [25]. However, the potential application of Hi-C technology in studying important economic traits or developmental processes in geese remains largely unexplored. In our laboratory, we constructed a reference genome for geese at the chromosome level, which serves as a solid foundation for investigating how chromatin spatially regulates gene expression in goose livers [13]. The mouse model is widely recognized as a highly relevant biomedical model for studying gene expression regulation. Previous studies have provided compelling evidence demonstrating that mouse livers' gene expression and dynamic chromatin interactions respond to an HFD [26, 27].

This study aims to explore the unique genetic mechanisms that enable goose livers to adapt to HFD-induced hepatic steatosis while avoiding the adverse effects commonly observed in mammals. By employing comprehensive multi-omics analyses, including Hi-C, ChIP-seq (histone H3K27ac), and RNA-Seq, we investigated the impact of HFD on the spatial conformation of chromosomes and gene expression in goose liver tissues. Additionally, conducting a cross-species comparative analysis with mice, focusing on one-to-one orthologous genes provides a comprehensive understanding of the adaptive responses to dietary stress in goose livers compared to other species. This study accentuates the potential parallels and distinctions between geese and mice in their physiological adaptability to the stressors introduced by HFD, offering novel insights into regulating fat deposition and hepatic fat management in response to excessive energy consumption.

## Methods

### Experimental animals

In a previous study [14], our lab collected normal and HFD-induced goose fatty liver tissues. The geese were kept in the same environment, provided with water, and fed the same diet. However, for the HFD group, a basic diet supplemented with the HFD was administered for 18 d [14]. Following the short-term feeding experiments, the entire cohort of geese subjects underwent euthanasia, and their respective liver tissues were meticulously collected, promptly subjected to flash-freezing in liquid nitrogen, and subsequently stored at –80 °C, all in preparation for subsequent assays.

A total of 200 female Kunming mice were obtained from the Animal Research Institute of the Sichuan Provincial People’s Hospital to generate the HFD-induced obese mouse model. In this study, the mice were randomly assigned to an HFD group consisting of 100 individuals (17.74 MJ/kg metabolizable energy, 11.26% crude protein, 6.8% fat, and 5% lysine) and a normal diet group consisting of 100 individuals (13.88 MJ/kg metabolizable energy, 15.37% crude protein, 2% fat, and 6.7% lysine). Under normal conditions, all mice were granted ad libitum access to food and water. Following a 16-week interval, a 12-h fasting period was instituted before the mice were sacrificed. Subsequently, a portion of the liver and diverse tissues were obtained from the mice, rapidly frozen in liquid nitrogen, and stored at –80 °C for subsequent experiments. Concurrently, the remaining liver tissues were submerged in 10% formalin and paraffin wax, undergoing subsequent dehydration and embedding in paraffin. The preparation procedures strictly adhered to the manufacturer's guidelines, and the specimens were subjected to staining using Hematoxylin and Eosin Staining Kits (C0107/C0109, Beyotime, Shanghai, China).

### In situ Hi-C library generation

A total of 10 geese (consisting of 5 HFD and 5 normal diet individuals) and 10 mice (including 5 HFD and 5 normal diet individuals) were chosen based on criteria that considered both liver weight and liver index. Individual Hi-C libraries were subsequently constructed for these 20 tissues [28]. The Hi-C libraries underwent an initial amplification of 8–10 cycles using a KAPA Hyper Prep Kit (Roche, KK8504, Wilmington, NC, USA) and were subsequently sequenced on the BGISEQ-500 platform.

### ChIP-Seq library generation

We conducted H3K27ac ChIP-seq following a protocol previously described for both geese and mice liver tissues [29]. Initially, we cross-linked 3–5 g of samples with DNA by treating them with formaldehyde at a final concentration of 1%. Following the initial cross-linking, the samples underwent two washes in cold phosphate-buffered saline buffer and two additional washes in cold water. We fragmented the chromatin using a sonicator to achieve DNA lengths between 200 and 500 bp. Throughout the procedure, we preserved the DNA input at –20 °C. We separated the soluble chromatin from the input DNA and performed immunoprecipitation using specific antibodies for H3K27ac (ab4729, Abcam, Cambridge, United Kingdom). Finally, we constructed sequencing libraries that were subsequently sequenced on the Illumina HiSeq X Ten platform.

### RNA extraction and RNA-seq library generation

RNA-seq data was generated for the 10 geese (5 HFD and 5 normal) and 10 mice (5 HFD and 5 normal) liver tissues used in Hi-C sequencing, including the RNA-Seq data of four geese (GSE119421) published in prior studies [14]. RNA extraction was conducted, involving the application of the Ribo-Zero^TM^ rRNA Removal Kit (RZH1046, Epicentre, Wisconsin, USA) to eliminate ribosomal RNA, and the subsequent purification of ribosome-free RNA through ethanol precipitation. Following this, the RNA underwent fragmentation using divalent cations in the NEBNext^®^ First Strand Synthesis Reaction Buffer (E7420S, NEB, Ipswich, MA, United States) (5×) at elevated temperatures. Ultimately, RNAseq libraries were constructed from the extracted ribosomal RNA (rRNA) and sequenced using the Illumina HiSeq X Ten platform.

### Hi-C data analysis

After eliminating low-quality nucleotides, contaminants, and adapter sequences, the high-quality Hi-C data from both the goose and mouse were aligned to the goose (ASM1303099v1 version) and mouse (mm39 version) reference genomes using the Juicer software [30]. Based on Knight-Ruiz and quantile algorithms, we generated contact matrices of various resolutions (100, 20, and 5 kb) and normalized matrices. The reproducibility evaluation in contact intrachromosomal matrices for both goose and mouse was conducted using the HiCRep software with default parameters [31].

We applied VNE to quantify the order in chromatin structure (100-kb resolution), provided that higher entropy corresponds to higher disease severity [32]. Principally, the correlation matrix is calculated as follows: C = corr(log_2_[A]) (A: Hi-C matrix of each autosome). Furthermore, the eigen decomposition of matrix C is computed, where *λ*1 ≤ *λ*i ≤ *λ*n are the eigenvalues of C, and the eigenvalues are normalized as follows :$\overline\lambda i=\frac{\lambda i}{{\sum }_{j=1}^{n}\lambda j}$. Finally, VNE is computed as follows: ${\text{VNE}}=-{\sum }_{i=1}^{n}\,{\overline\uplambda} i{\text{ln}}(\overline{\uplambda }i)$.

### Identification of Compartments A/B

At a 20-kb resolution, principal component analysis was performed on matrices, generating PC1 vectors for each chromosome in every sample. Subsequently, Spearman's correlation between PC1 and genomic characteristics was calculated. Referred to as Compartment A were bins demonstrating a positive Spearman’s correlation, while those exhibiting a negative correlation were defined as Compartment B. The A-B index, representing the likelihood of genomic segment interdependence with either Compartment A or B, was estimated at a resolution of 20 kb. These definitions are based on the previously described 100 kb resolution. Based on the 20-kb resolution matrices, we identified the Compartments A/B using this method [33].

### Topologically associated domains (TADs)

By subtracting or adding ten bins from the center of each bin, the directionality index (DI) was initially calculated with a resolution of 20 kb. Further, the hidden Markov model was used to determine the state of DI at the TAD border. Upon identifying the large TAD using DI, we employed the minimal insulation score (IS) and its normalized IS vector to partition the large TAD into smaller TADs [32]. To further determine whether the HFD induced changes in TAD and TAD boundaries, we assessed the TAD structures of the goose genomes at a resolution of 20 kb using the DI [34] and the IS [35] algorithms.

### Analysis of promoter–enhancer interactions (PEIs) for genes

A 5-kb resolution contact matrix was generated by pooling Hi-C reads from five biology replicates to identify PEIs in goose and mouse. We identified overrepresented interactions within the promoter region PSYCHIC software [32, 36]. Applying the PSYCHIC algorithm to their ultra-deep Hi-C contact maps, we utilized it to identify a repertoire of long-range PEIs at a resolution of 5 kb [32]. We retained PEIs with false discovery rates (FDRs) < 0.05 and interaction distances < 20 kb. A regulatory potential score (RPS) was calculated for each gene to understand dynamical rewiring better. The RPS was calculated as ∑n (log_10_ In), which indicates the normalized interaction intensity (observed value − expected value). An enhancer without promoter interaction indicates a zero RPS. In investigating RPS genes with low RPS, we observed that even minimal fluctuations in their expression led to significant fold changes (FC). Therefore, to identify meaningful changes, we set the criteria of log_2_FC > 2 and delta > 3.

### Analysis of ChIP-Seq

Mapping the high-quality ChIP-seq data to the goose and mouse genomes was achieved using the Burrows-Wheeler Aligner (BWA) (v 0.7.15) [37]. Subsequently, potential PCR duplicates were filtered using Samtools (v 1.3.1) software [38]. Following merging the bam files of biological replicates, we identified H3K27ac (an indicator of active enhancers) peaks using the SICER (v 1.1) [39], which identifies peaks with a cutoff of *FDR*-value < 0.05. Retaining H3K27ac ChIP-seq peaks from the merged sample and at least one biological replicate, we subsequently merged and ranked these peaks in neighboring enhancer elements (within 12.5 kb) to identify the signal. The signal strength per 1 kb bin was then calculated using a specific formula: log_2_ (IP FPKM/input FPKM). By identifying H3K27-enriched regions by ChIP-Seq analysis, distal interaction regions distant from the promoter were classified as poised enhancers (PE), regular enhancers (RE), or super-enhancers (SE) using the standard Rank Ordering of Super-Enhancers (ROSE) algorithm [40]. The data were further visualized using the Integrative Genomics Viewer (version 2.12.2) browser [41].

### Analysis of RNA-seq data

We removed the adaptors, low-quality sequences, and sequences of < 50 bases using an in-house pipeline and then mapped the RNA-Seq data to goose and mouse genome sequences following the standard RNA-Seq pipeline parameters of ENCODE using STAR (version 2.7.0c) software [42]. Transcripts for each library were filtered based on length (250 bp), FPMK expression level (0.1), clipped exons (15 bp), and background noise reduction using the Assemblyline. We merged all filtered libraries, compared them with reference annotation, and removed transcripts annotated as protein-coding genes (PCGs). The differential gene expression analysis between groups was calculated using edgeR software (v 3.22.5) [43]. To identify the differential expression genes, threshold values of FDR < 0.05 and |log_2_FC|≥ 1.5 were used as cutoff values for the analysis. To create RNA-Seq signal graphs, Samtools was used to analyze BAM files and determine read numbers for each window, and the data were further visualized using the Integrative Genomics Viewer (version 2.12.2) browser [41].

### Cross-species network analysis

We used the OrthoMCL software [44] to compare the orthologous genes between goose and mouse. Subsequently, only the single-copy orthologous genes shared between the two species were selected for further analysis.

## Results

### Dynamic chromatin architecture and transcriptome induced by HFD in goose liver tissues

Feeding an HFD for 18 d resulted in a 37.50% increase in goose body weight (normal 4.50 kg, HFD 6.09 kg, foldchange = 1.38, *P* = 1.22 × 10^–3^), the liver index increased 2.73-fold (normal 1.32%, HFD 3.61%, *P* = 5.69 × 10^–3^), and the liver content increased 9.15-fold (normal 11.51 mg/g, HFD 105.35 mg/g, *P* = 5.31 × 10^–6^). The liver from the HFD group exhibits substantial yellowing, along with the deposition of lipid droplets, compared to the dark red livers in the normal group [14].

**Fig. 1:**
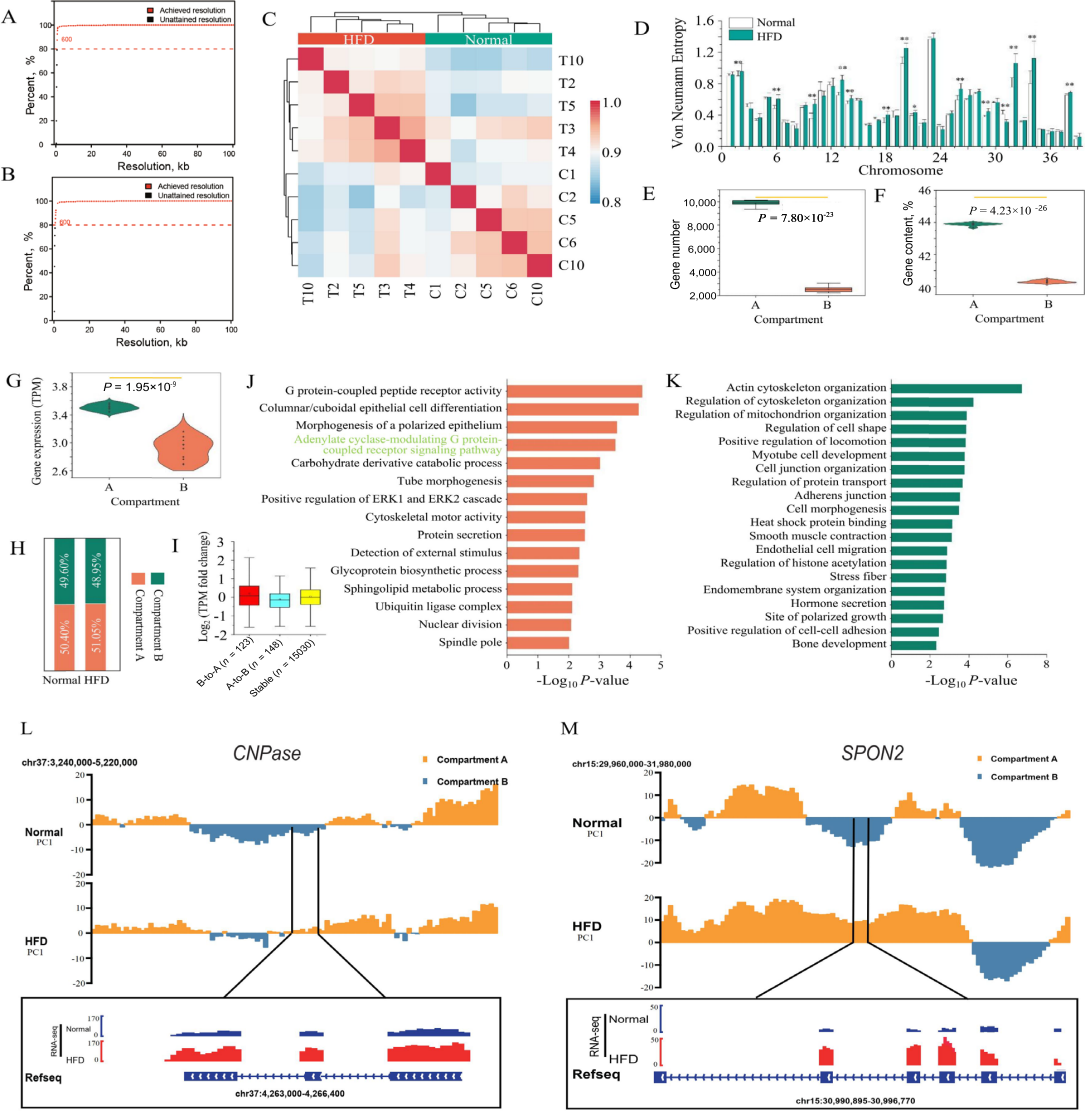
Compartmentalization dynamics in goose liver tissues induced by HFD. The goose HFD (**A**) and normal liver (**B**) group Hi-C data access resolution; **C** The Heatmaps of the HiCRep correlation coefficients of the goose samples; **D** Von Neumann entropy (VNE) measures the degree of disorder in chromatin structure; The gene number (**E**), GC content (**F**) and gene expression (**G**) of Compartments A and B; **H** The proportion of Compartments A and B in goose genome; **I** The gene expression of the three types of goose Compartments. The functional analysis of the goose genes embedded in regions that experience the B-to-A (**J**) A-to-B (**K**)

To investigate the effect of HFD on chromatin architecture and gene expression in goose fatty liver, we collected 10 goose liver samples (5 normal, 5 HFD) to generate 10 Hi-C and 10 RNA-seq data. The Hi-C analysis yielded approximately 6.22 billion valid contacts for both the HFD and normal diet groups of geese (622.31 million contacts per library) ( Fig. S1, Table S1). By merging the intrachromosomal contacts of the HFD and normal diet groups, we achieved a maximum resolution of 600 bp (Fig. 1 A–B). At 100-kb resolution, the Hi-C maps obtained from the replicates of the HFD and normal diet groups showed high reproducibility in geese (Fig. 1C). Interestingly, we observed that the whole chromatin architecture was stable induced by the HFD (Fig. 1D). Moreover, we obtained a total of 143.39 Gb of high-quality RNA-Seq data from 10 liver tissues. In total, 15,737 genes exhibited evident expression. Their expression levels were found to be highly reproducible within the biological replicates (Fig. S2A) and identified 1,361 differentially expressed genes (DEGs) between the HFD and normal diet groups (Fig. S2B, Table S2). Among the DEGs, 865 up-regulated genes involved in various signaling processes, energy production and consumption, and metabolic pathways, including the mitotic cell cycle, oxidoreductase activity, alpha-amino acid metabolic process, and carbon metabolism (Fig. S2C, Table S3). On the other hand, 496 DEGs were down-regulated and associated with pathways related to growth, development, and cancer, such as pathways in cancer, as well as responses to hormones, including insulin and peptide hormones (Fig. S2D, Table S3). The results emphasize the dynamic changes in phenotypic traits, chromatin architecture, and transcriptome caused by HFD in goose liver tissues.

### Compartmentalization A/B switch and the dynamics of and TAD boundaries induced by HFD in goose liver tissues

We analyzed multivariate entropies utilizing the VNE index to investigate the influence of an HFD on the chromatin structure within goose liver. Notably, the observations revealed a substantial variance in VNE values between the HFD-induced liver tissues and the normal liver tissues (Fig. 1D). This discrepancy strongly suggests the presence of a disorganized and more relaxed chromatin architecture in geese liver induced by the HFD. Based on the 20-kb resolution contact map, we observed that Compartment A, characterized by higher GC content, gene expression, and gene number, exhibited significant differences from Compartment B (Fig. 1E–G). Additionally, we found that 51.05% and 50.40% of the entire genome consisted of Compartment A bins in the HFD and normal goose liver, respectively (Fig. 1H). Subsequently, we identified 23.80 Mb (2.42% of the goose genome) of compartmental switching between the two groups. We found that the rearrangement of compartmentalization significantly contributed to the changes in gene expression induced by HFD, as measured by the abundance of messenger RNA (Fig. 1I). The gene expression switching induced by HFD exhibited predominantly unidirectional changes, suggesting an association between dynamic compartmentalization and subtle alterations in gene expression (Fig. 1I). We observed that genes undergoing an A-to-B switch (148 genes, 18 DEGs) (Fig. 1J, Table S4) were primarily involved in "actin cytoskeleton organization" (GO:0030036, 12/538, *Q* = 4.13 × 10^–3^), and "regulation of cytoskeleton organization" (GO:0051493, 9/531, *Q* = 2.66 × 10^–1^) (Fig. 1K, Table S5). These genes include *DEMA* (maintaining glucose homeostasis), *SELENOM* (involved in hepatic steatosis, inflammation, lipid metabolism, and fatty acid oxidation, is associated with hepatocellular degeneration and carcinoma), *EDEM3* (controls the uptake of very low-density lipoprotein and plasma triglycerides), and *ABCAA* (involved in lipid metabolism). Specifically, *ERRγ* modulates the activity of genes associated with energy metabolism, *PANX2* (controls glucose tolerance and apoptosis), *LGR6* (a key component of HCC survival), and *CNPase* (a gene that could have a crucial role in upholding mitochondrial function and averting mitochondrial malfunction) (Fig. 1L, Table S5). Conversely, the genes in the dynamic compartmentalization region that experienced a switch from B-to-A (123 genes, 11 DEGs) (Fig. 1I, Table S4) were mainly enriched in "carbohydrate derivative catabolic process" (GO:1901136, 4/170, *P* = 9.53 × 10^–4^, typically *GLUT8*), "glycoprotein biosynthetic process" (GO:0009101, 4/266, *P* = 4.81 × 10^–3^, *NPY1R*) (Fig. 1I, Table S5). These genes are involved in metabolic processes (*GLUT8*, *TAp63*, *RIMKB* and *ANR22*) and immune processes (*NPY1R*, *ACKR3*, *SPON2*, *Cause* and *VAT1*) (Fig. 1M).

**Fig. 2:**
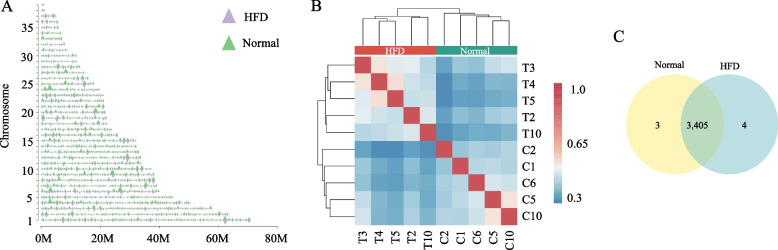
The boundaries of TADs exhibit significant stability within the goose liver in response to an HFD. **A** The distribution of TADs in both high-fat diet (HFD) and normal goose liver tissues; **B** The overlapping TAD boundaries between the groups of goose liver tissues exposed to a high-fat diet (HFD) and those with normal conditions; **C** The Spearman’s *r* heatmap of the insulation score (IS) in goose HFD and normal liver tissues

In the goose genome, 2,019 TADs (length 439.02 kb, median 400.00 kb) and 1,894 TADs (length 461.93 kb, median 404.00 kb) were identified in the HFD and normal liver tissues, respectively (Fig. 2A). The TAD boundaries generated by both algorithms exhibited remarkable consistency across biological replicates in TAD contacts (Spearman’s* r* > 0.80) (Fig. 2B). We only found 7 TAD boundaries (4/3,409 in HFD, 3/3,408 normal) altered by HFD (Fig. 2C, Table S6). The results indicate that within the dynamic TAD boundaries in goose liver tissue, there was an observed gain in the *GK* (glycerol kinase) gene. In contrast, the *PRODH* (proline dehydrogenase 1) gene experienced a loss at the boundary in response to an HFD (Table S6). These observations indicated that the goose genome TAD and TAD boundaries were highly stable and less affected by the HFD.

### The rewiring of PEI in goose liver tissues induced by HFD

**Fig. 3:**
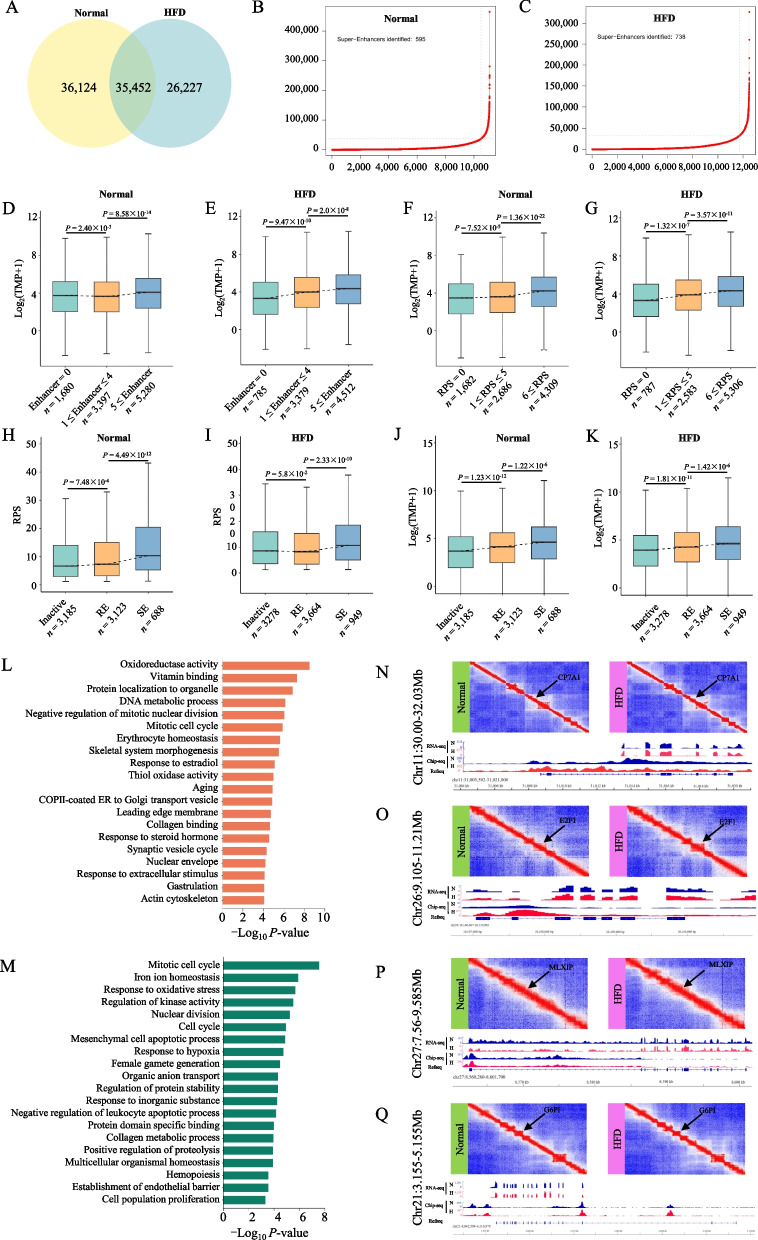
The induction of an HFD leads to the rewiring of PEI in the goose liver. **A** The common and specific PEI in goose normal and HFD liver; Determination of super-enhancers (SEs) by ranking H3K27ac signals using the ROSE algorithm in geese normal (**B**) and HFD (**C**) liver tissues; The number of enhancers and their impact on gene expression were determined for normal (**D**) and HFD (**E**) liver tissues of geese; The RPS value and its association with gene expression were examined in normal (**F**) and HFD (**G**) liver tissues of geese; The RPS value was investigated in SE, RE, and SE regions of normal (**H**) and HFD (**I**) liver tissues of geese; The impact of SE, RE, and SE regions on gene expression was analyzed in normal (**J**) and HFD (**K**) liver tissues of geese. The function and pathway enrichment analyses for the gene increased (**L**) or decreased (**M**) significantly RPS in goose HFD liver tissues. PEI rewiring of the functional gene *CP7A1* (**N**), *E2F1* (**O**), *MIXIP* (**P**), and *G6PI* (**Q**)

Since the spatiotemporal chromatin architecture facilitates or constrains PEIs to dynamically regulate gene expression, we assembled comprehensive genome-wide PEIs in goose liver tissues exposed to an HFD. We identified 61,679 and 71,576 PEIs in the goose HFD and normal liver tissues, respectively, and 35,452 PEIs were shared between the two groups (Fig. 3A). By the distribution of the H3K27 acetylation (H3K27ac) peaks, enhancers can be categorized as SE, RE, or PE (Fig. 3B, C). In the goose genome, genes regulated by multiple enhancers demonstrated a simultaneous increase in transcription, indicating that enhancers affect target genes in an additive manner (Fig. 3D, E). To accurately determine the dynamic rewiring of PEIs induced by the HFD, we calculated the RPS of each gene to examine the regulatory effects of multiple enhancers on specific genes. The expression of genes exhibiting higher RPS levels was found to be significantly elevated compared to genes with lower RPS levels (Fig. 3F, G), indicating that the relative contribution of each enhancer to a gene's expression was primarily influenced by its effect divided by the total effect of all enhancers. As anticipated, genes associated with SEs exhibited markedly higher levels of RPS and gene expression than genes associated with REs (Fig. 3H, I).

Furthermore, the expression of genes linked to SEs was significantly higher than that of genes associated with PEs (Fig. 3J, K). These results emphasize enhancers' essential and discriminating function governing gene expression across various activities. To investigate the potential impact of extensive rewiring of PEIs, we conducted a comparative analysis of RPS for each gene between HFD and normal liver tissues in geese. This analysis identified genes exhibiting significant differences in RPS, shedding light on the potential mechanisms underlying these observed changes.

By integrating the rewiring events of PEIs in geese from both the HFD and normal groups, we identified 3,387 genes with significantly differential RPS (|log_2_FC|> 3 and |Δ|> 2) between HFD and normal conditions. Among these, 2,396 genes exhibited a substantial increase in RPS in the HFD group, while 991 genes showed a remarkable increase in the normal group (Table S7). The significantly increased RPS in goose HFD was enriched in oxidoreductase activity, essential cellular functions (e.g., cellular response to DNA damage stimulus, protein localization to organelle, regulation of kinase activity), and the control of cell growth and differentiation (e.g., cell population proliferation, regulation of neuron apoptotic process). In addition, we observed a significant decrease in RPS in the goose HFD group. The downregulated RPS was overrepresented in categories associated with oxidative stress response, cell population proliferation, cell differentiation, and development. Specifically, we observed enrichment in epithelial cell differentiation, epithelial cell development, endothelial cell development, biological regulation, and growth-related processes. These findings suggest that the goose liver undergoes enhanced energy consumption processes and experiences various disorders due to metabolic stress, leading to altered cell growth and differentiation (Fig. 3L, Table S10). Furthermore, our results highlight the central role of the goose liver in metabolic homeostasis response to lipid overload and its association with diverse disorders caused by metabolic stress. Typically, *DHB12* and *FADS1* (fatty acid metabolism), *FABP7* and *FADS1* (PPAR signaling pathway), *DHB12* (fatty acid elongation), *AKT3* (glucagon signaling pathway and insulin resistance), *CP7A1* (Fig. 3N), *E2F1* (NAFLD disease) (Fig. 3O), *MIXIP* (HCC disease) (Fig. 3P) *G6PI* (Fig. 3Q) and *LDHA* (glycolysis/gluconeogenesis) shown more PEIs and were regulated by the SE or RE in goose HFD tissues (Table S8). Interestingly, some energy metabolism genes with significantly decreased enhancers were regulated by SE or RE, including *AL7A1* ("Fatty acid degradation" and "Glycerolipid metabolism"), *PLCA* ("Glycerophospholipid metabolism" and "Glycerolipid metabolism") and *PLIN1* (PPAR signaling pathway) (Fig. 3M, Table S8).

To enhance our comprehension of the rewiring of PEIs in response to an HFD in goose liver, we assessed the PRS profiles of the liver between goose fed a normal and HFD diet. A comprehensive set of six representative candidate gene sets, including amino acid metabolism (52 out of 241 genes), glucose metabolism (18 out of 84 genes), fatty acid metabolism (137 out of 613 genes), tricarboxylic acid cycle (TCA) components (29 out of 41 genes), bile acid metabolism (4 out of 30 genes) and drug metabolism (19 out of 130 genes) displayed significant increases or decreases in RPS of within goose HFD liver tissues. Interestingly, genes with increased RPS in goose HFD liver tissues included *NQO1* (amino acid metabolism), *FADS1, FADS2* (fatty acid metabolism), *G6PI*, *HGF* (glucose metabolism), *ACS2L*, and *FAS* (glucose metabolism and tricarboxylic acid cycle). All of these genes showed that the 3D genome regulated the genes involved in the metabolism.

To gain insight into the genomic reorganization response associated with metabolic adaptation in geese, we comprehensively analyzed alterations in RPS profiles for distinct gene sets, encompassing immunity, HCC, and NAFLD. Interestingly, among the 44 immunity-related genes, we identified five genes that exhibited noteworthy changes. Our investigation of HCC signatures revealed that nine genes were associated with the less aggressive HCC subtype (S-I, good prognosis, *BODG*, and *ABCA5*). Furthermore, our analysis indicated a decrease in the expression of four genes within the same HCC subtype (S-II), typically involving pivotal genes, such as *HNF4*α and *HNF1*α. A significant subset of the 68 signature genes displayed predominantly heightened RPS, especially concerning the more aggressive HCC subtype (S-III) associated with a poorer prognosis. Remarkably, key genes such as *CATC* and *IKBE* were prominently enriched within this subset. Among the 126 genes implicated in NAFLD (ko04932), 23 genes demonstrated significant RPS modifications between the compared groups, including the key genes *BID*, *IGF1R*, and *INS.* These findings indicate potential regulatory functions for the genes involved in immunity, HCC, and NAFLD, underscoring their putative roles in the intricate processes governing these biological phenomena.

### Chromatin architecture changes in the mouse liver in response to HFD-induced obesity

**Fig. 4:**
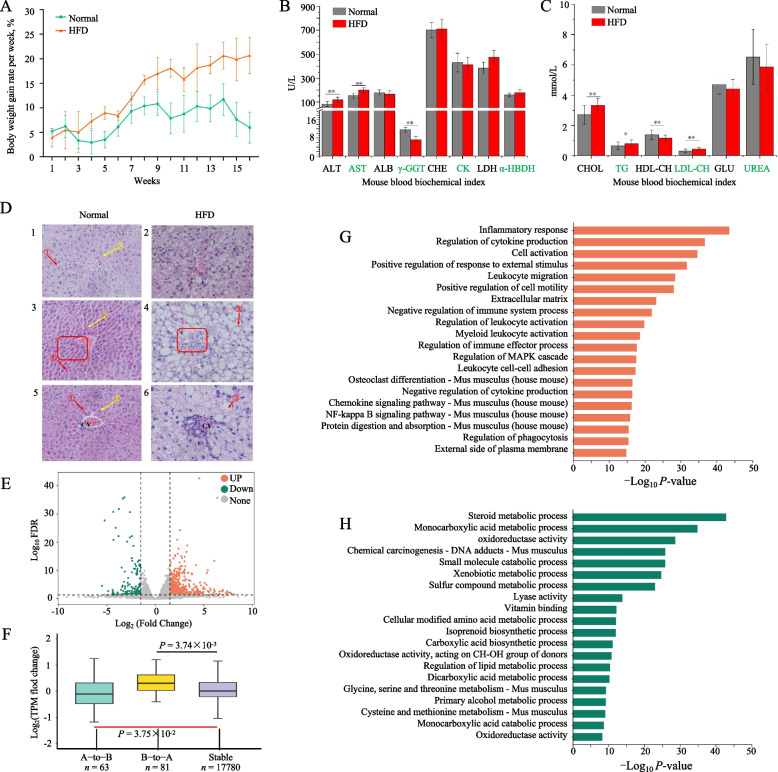
HFD altered mouse liver tissue weight, blood, gene expression, and liver Compartments. **A** The mice were exposed to an HFD for 112 d, and their weight gain reached a plateau without any further significant increase. This prolonged exposure to the HFD led to the development of an inflammatory physiological state in the mice, as evidenced by a significant increase in serum levels (**B, C**). **D** The HE staining demonstrated the accumulation of intracellular lipids, ballooning, and signs of inflammation. **E** The differentially expressed genes (DEGs) in the liver of mice exposed to a high-fat diet (HFD) compared to a normal diet. **F** The gene expression of the three types of mouse Compartments. The gene enrichment analysis was conducted to investigate the functional characteristics of the genes located in regions that undergo the transition from state B to state A (**G**) and from state A to state B (**H**)

We employed the mammalian biomedical model mouse as the experimental model to investigate the distinctive characteristics of goose livers in response to excessive energy intake and compare them with their mammalian counterparts. The mice were fed an HFD for 112 d until their weight gain plateaued without further significant increase (Fig. 4A). Interestingly, the goose gained more body weight (goose 37.5% vs. mouse 20.62%), liver weight (goose 3.82 vs. mouse 2.20), liver index (goose 2.73 vs. mouse 1.85), and TG content of liver tissues (9.15 vs. 4.26) on an HFD than the mouse. Moreover, a notable rise in serum concentrations of ALT, AST, GGT, CHOL, HDL-CH, and LDL-CH (Fig. 4B, C), indicated potential liver dysfunction and altered lipid metabolism due to the HFD. Additionally, histological staining using hematoxylin and eosin (HE) demonstrated the accumulation of intracellular lipids, ballooning, and inflammation in the liver (Fig. 4D). In conclusion, this prolonged exposure to the HFD induced an inflammatory physiological state in the mouse.

To explore the characteristics of chromatin structure and gene expression in the mouse liver induced by the HFD, we selected 10 liver tissues (5 HFD and 5 normal) to perform the Hi-C and RNA-seq sequencing. In the mouse liver tissues of the HDF and normal diet groups, we obtained 132.06 Gb of clean RNA-Seq data (13.21 Gb per sample) (Table S9). We identified a total of 1,483 differentially expressed genes (DEGs) between the HDF and normal groups, meeting the criteria of FDR < 0.05 and |log_2_FC|> 1.5 (Fig. 4E, Table S10). For the 3D mouse genome, the mouse HFD and normal diet groups yielded about 6.16 billion valid contacts (615.55 million contacts per library) (Fig. S3, Table S11), and the intrachromosomal contacts were merged to achieve a maximum resolution of 2,000 bp in the mouse (Fig. S4). In mice, the maps obtained from replications of HFD or normal diet groups of geese were highly reproducible at 100 kb resolution (Fig. S4).

**Fig. 5:**
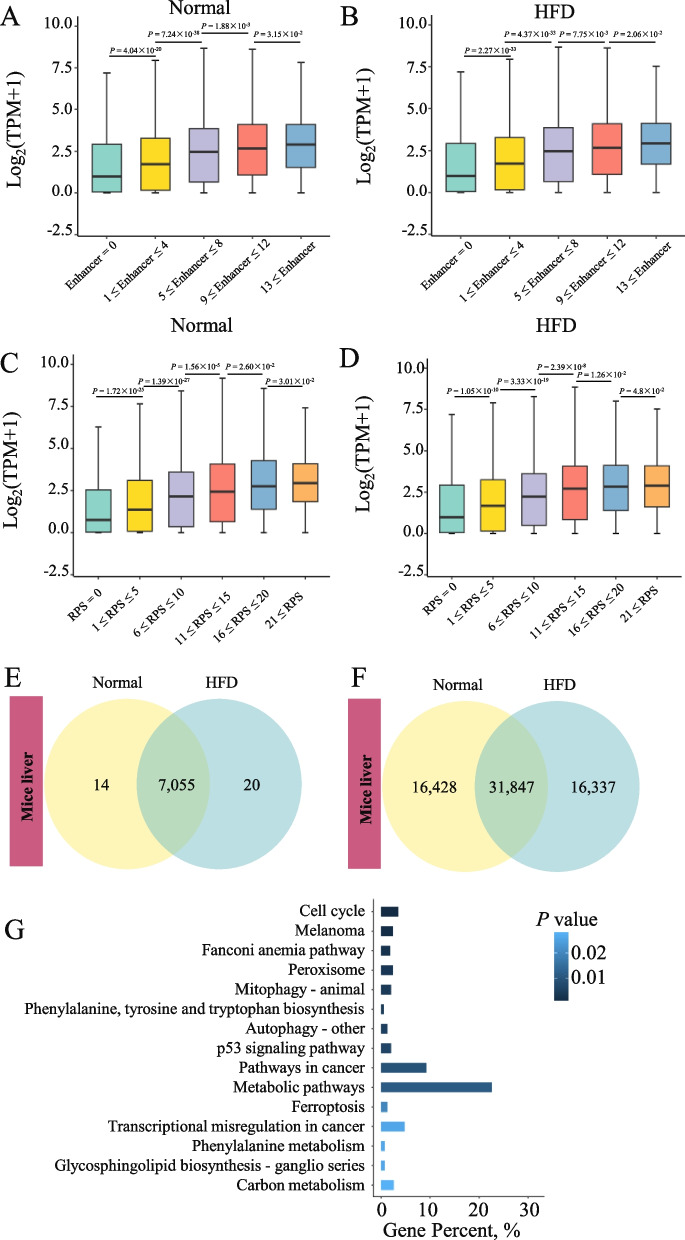
The effect of HFD on TAD and PEI changes in mouse liver tissues. The study investigated the determination of enhancer quantity and their effects on gene expression in mouse liver tissues under two conditions: normal (**A**) and high-fat diet (HFD) (**B**). Additionally, the examination of RPS value and its correlation with gene expression was carried out in mice's normal (**C**) and HFD (**D**) liver tissues. Moreover, identifying shared and distinct TAD boundaries (**E**) and exploring PEI (**F**) were conducted in normal and HFD mouse liver tissues. **G** Gene functional analysis of goose-specific genes

At the compartmental level, the genes embedded in A-to-B switched regions (63 genes, 14 DEGs) (Fig. 4F, Table S12). A switch from B-to-A was experienced by 81 genes within the dynamic compartmentalization region (1.20%) (Fig. 4F, Table S12). Interestingly, the DEGs within dynamically compartmentalized regions shift from a B-to-A state, displaying significant enrichment in processes related to inflammation and signaling pathways (Fig. 4G, H). At the TAD level, we identified 4,480 TADs (length 460,498 bp, median 400,001 bp) in normal groups and 4,511 TADs (length 458,366.6 bp, median 400,001 bp) which were highly reproducible within biological replicates in TAD contact (Spearman’s *r *> 0.80) (Fig. S5, S6). We identified 14 TAD boundaries with 13 genes in the normal diet group and 20 TAD boundaries with 19 genes in the HFD group (Table S13). Most of the genes identified in the normal group are predicted genes, and limited studies have been conducted on these genes, namely *DEFA36*, *MUP17*, *PRAMEL35*, *VMN1R122*, and *DEFA33*. The observed phenomenon is attributed to the inherent stability and organization of the genome’s TAD chromosome architecture in both goose and mouse liver tissues, which appears resistant to an HFD effect. Consistent with the findings in the goose genome, the influence of enhancers or RPS on target genes follows an additive pattern (Fig. 5A–D). At the PEI level, we identified 1,124 significantly different PEIs with 203 DEGs between mouse HFD and normal liver tissue (|log_2_FC|> 3, |Δ|> 2) accompanied by changes in enhancer activity (Fig. 5E, F, Table S14). Similar to the functional analysis of the DEGs (Table S15), the genes within the dynamic compartmentalization region switch from B-to-A (Table S16), and the genes were significantly enriched in the process involved in the inflammatory and signal processes (Table S17).

### Evolutionary divergence of local spatial context in goose liver tissues

We investigated the genes with significantly different RPS diverges between the two species. To this end, we used OrthoMCL software to identify conserved and species-specific variations induced by HFD. Of the 6,988 identified one-to-one orthologous correspondence between the goose and mouse, we found that 1,919 genes (27.88%) define an orthologous set of genes regulated by Compartments or PEIs shared by the two species. Among the regulated genes, a substantial proportion showed species-specific regulation, with 1,467 genes (76.45%) being goose-specific and 325 genes (16.94%) being mouse-specific, regulated by Compartments or PEIs. Additionally, 124 genes (6.46%) were shared between goose and mouse, regulated by PEIs in either species (Table S18).

To gain insights into the distinctive characteristics of lipid deposition capacity and the liver's tolerance to hepatic steatosis, we specifically examined the subset of 1,467 genes regulated by the unique 3D genome architectures in geese. The goose-specific genes were enriched in the process “metabolic pathways” (Fig. 5G, Table S19), including "fat digestion and absorption (6 genes; typically, *APOA4*)", " cholesterol metabolism (7 genes; typically, *APOC3*, *CYP7A1*)", "PPAR signaling pathway (7 genes; typically, *FABP2*, *FABP6,* and *FABP7*)", and "oxidative phosphorylation (14 genes; typically, *NDUFA5*, *NDUFB10*, *NDUFB6*, *NDUFS8*, and *NDUFV1*)", indicating that goose fat metabolism differs from that of mouse. The goose-specific genes were enriched in the process "Pathways in cancer", including Insulin resistance (6 genes; typically, *MLXIP*), Nonalcoholic fatty liver disease (10 genes; typically, *NDUFB10* and *MLXIP*), HCC (11 genes; typically, *IGF2* and *E2F1*); NF-kappa B signaling pathway (12 genes; typically, *LY96*). These findings functionally explain the unique regulatory mechanisms of goose liver in response to HFD.

Of particular interest is the observation that of the cohort of 127 shared genes, 54 exhibited contrary regulatory trends in the context of three-dimensional genomic structural modulation. These genes predominantly congregate within functional categories such as amide biosynthetic processes (GO:0043604), including *ASAH1*, *ST8SIA4*, *RNF14*, *RSL24D1*, and *MRPL16*. Additionally, they manifest a presence within the oxidoreductase complex and cytokine binding (GO:1990204), involving constituents such as *NCF4*, *NDUFA8*, *WDR93*, *MRPL16*, *SLC25A29*, and *PLIN1*, as well as cytokine binding (GO:0019955) instances, encompassing entities such as *CNTFR*, *CHRDL2*, and *IL20RA*. Notably, these genes regulate diverse physiological processes, including immune responses, inflammatory reactions, cellular proliferation, differentiation, and apoptosis.

We identified the expansion and contraction in the goose genome in a previous study [13]. A total of 48 genes were identified within 839 expanded gene families. Notably, we uncovered the existence of *FAS*, *ABCA5*, and *BMP1*. Additionally, among the 52 positively selected genes, eight displayed significant variations in RPS, including a significant increase in *GCH1* and *NPY5R*. In conclusion, both the expansion and positive selection of genes are poised to play pivotal roles in the adaptive mechanisms of liver metabolism, especially compared to other avian species.

## Discussion

### The goose supplied an ideal animal model for liver disease

In this study, the goose liver tissues subjected to an HFD displayed metabolic dysfunction with significant phenotypic variation with increased body weight, liver weight, liver index, and liver tissue TG content compared to HFD-induced mice manifesting evident metabolic dysfunction, which suggests that domestic geese might possess resilience against NAFLD and HCC, even in the presence of obesity, potentially establishing them as a valuable animal model for human liver disease. To investigate the unique genetic characteristics of goose liver tissues, we thoroughly examined multi-omics data (Hi-C, H3K27ac-ChIP-Seq, and RNA-Seq) from goose liver tissues induced by an HFD to explore the unique protective mechanism in response to HFD.

This goose fatty liver induced by HFD is similar to the pathogenesis of NAFLD [2, 45, 46]. Animal models of NAFLD are predominantly established using high-fat dietary feeding, pharmacological intervention, and genetic modification. These models encompass a diverse range of mammals, spanning monkeys [47], mice [48], rats [49], pigs [50], rabbits [51], and geese [46], with each model offering distinct advantages and holding specific applicability. The goose HFD liver is significantly yellow compared to the dark red liver of the normal-diet group, and lipid droplet deposition has previously been reported in goose livers [52, 53]. Previous studies conducted in our lab or by other researchers have demonstrated that HFD leads to a significant increase in the concentrations of CHOL, ALT, AST, GGT, TG, and HDL in goose plasma, as well as an overall increase in total lipid content compared to normal liver tissue [53, 54]. Similar to the goose, the mouse was also led to obesity but with an inflammation physiological state by the HFD, which was not sufficient for triggering hepatic ERS or NAFLD. The unique lipid metabolism mechanism in geese, enabling efficient fat accumulation in the liver and positioning them as an ideal model for studying non-alcoholic fatty liver disease (NAFLD), not only enhances our understanding of lipid metabolism but also reveals protective responses to overfeeding, offering novel strategies for increasing goose fatty liver production and preventing fatty liver disease in other species [5, 6]. Moreover, the physiological capability of significant post-overfeeding enlargement in goose fatty liver without evident pathological symptoms indicates the presence of protective mechanisms, making it a distinct model for fatty liver research and promising innovative approaches to enhance goose fatty liver production and avert fatty liver disease across various species [45, 46].

### Chromatin and transcriptomic changes triggered by HFD in goose liver tissues

Overall, our findings illustrate that alterations in gene expression in goose liver tissues align with changes in the A/B Compartments, TADs, or PEIs, suggesting that the 3D genome architecture structure (Compartments, TADs, or PEIs) plays a relatively conserved role in gene regulation. In the transcriptional landscape, consistent with previous research in our lab [14], the up-regulated differentially expressed genes (DEGs) are implicated in energy production, consumption, and metabolic pathways. Conversely, the down-regulated DEGs are linked to pathways associated with cancer or immune responses, in alignment with previous findings, such as *SCD1*, *ELOV2, FADS1, FADS2* [55], and *BMP6* [56]. Based on prior research [13, 14], we upgraded the goose genome sequence and incorporated six new RNA sequencing datasets out of ten, enhancing both the quality of our RNA sequencing data and the robustness of our identification methodology.

### Metabolic adaptations in goose liver to HFD: insights from 3D genomic structure

The HFD induces lipid deposition in goose fatty tissue due to an imbalance where triglyceride TG production surpasses apolipoprotein transport capacity, and fatty acid overproduction exceeds β-oxidation [57]. In this study, the HFD induced the key genes with significantly different RPS values and enhancer numbers by being involved in the synthesis of long fatty acid metabolism. For instance, *FASN* is crucial in converting glucose into lipids, serving as a central determinant for the hepatic capacity to synthesize fatty acids and functioning as a key regulator in hepatic de novo lipogenesis [58], which is regarded as a promising therapeutic target for both NAFLD and HCC [59, 60]. Mouse with liver-specific *FASN* knockout exhibited hypoglycemia and fatty liver, both of which were ameliorated upon the introduction of a dietary fat regimen [61]. In cross-species research, *FASN* exhibits distinct 3D expression regulation in mouse and goose genomes. Consequently, this may be one of the contributing factors to the distinctive lipid metabolism characteristics observed in goose liver tissue. Moreover, *FADS1* and *FADS2* are two rate-controlling enzymes in de novo fatty acid synthesis and TG metabolism, and the two genes are potential targets therapeutic targets for NAFLD disease [62, 63], serving as pivotal fatty acid desaturases in the process of de novo lipogenesis and ensuing steatosis within goose liver [64]. The knockdown of *FADS1* in the HepG2 cell line resulted in a notable reduction in cellular levels of LC-PUFAs. Concurrently, it induced an elevation in lipid accumulation and the formation of lipid droplets, accompanied by significant modifications in diverse pathways associated with lipid homeostasis. Furthermore, it remarkably impacted fatty acid oxidation [65]. Similarly, in *FASD2* knockout (FADS2^-/-^) mice, hepatic triacylglycerol and cholesterol accumulation are significantly exacerbated when exposed to a diet deficient in polyunsaturated fatty acids [66]. *HSD17B12* (17-beta-hydroxysteroid dehydrogenase type 12) plays a pivotal role in the synthesis of very-long-chain fatty acids, elongation of long-chain fatty acids, as well as lipid metabolism and metabolic homeostasis in the liver [67]. Mice lacking *HSD17B12* exhibit fatal systemic inflammation and lipolysis, decreased numbers and sizes of lipid droplets, microsteatosis, and increased TG accumulation [68], making it a potential target for NAFLD [69]. *OGDH*, as one of the rate-limiting enzymes in the TCA, plays a pivotal role by catalyzing an irreversible process within this metabolic pathway [70], which is linked to conditions such as developmental delays, hypotonia, movement disorders, metabolic disturbances, obesity, and diabetes [71]. A previous study demonstrated that *OGDH* silencing accelerates HCC progression via glutamine metabolism reprogramming, highlighting *OGDH* as a promising biomarker and therapeutic target for HCC [72].

### TADs are stable in the goose or mouse genome induced by an HFD

At the TAD level, we updated the analysis pipeline and identified more TAD structures (number: 1,894; size: 461 kb) in goose and mouse liver tissues than those identified in the previous study by our lab [13], and the TAD structures are consistent with those in the chicken and pig genomes [19, 22]. A previous study showed that TADs are stably induced by HFD in mice [27]; we identified that the switch TAD boundaries between the two groups exhibited limited annotation of prominent genes. Moreover, a majority of the annotated genes were predicted or anticipated, with limited existing research. Notably, goose and mouse liver tissues demonstrated a stable and ordered genome TAD chromosome architecture, which remained unaffected by the HFD. Interestingly, in goose liver tissue, dynamic TAD boundaries showed a gain in *GK* and a loss in *PRODH* in response to an HFD. *PRODH*, located on the inner mitochondrial membrane, is crucial for proline metabolism and energy synthesis in proline metabolism, energy synthesis, and stress response [73, 74]. Moreover, *PRODH* significantly impacts cancer progression through its dual role in ATP production and ROS generation, acting as both a tumor suppressor and oncogene, making it a critical therapeutic target for HCC [75, 76]. The *GK* gene, crucial for glycerol metabolism, produces glycerol kinase that transforms glycerol into glycerol-3-phosphate, a key process underpinning triglyceride backbone formation and is essential for energy production, glycerolipid synthesis, regulation of energy, lipid composition, and signal transduction [77]. Disruptions in the *GK* gene can lead to metabolic disturbances, emphasizing its critical importance in health preservation and disease intervention. In the context of liver cancer, changes in GK activity can be detected at the disease's early stages and continue throughout its advancement, which suggests that the *GK* gene is a potential therapeutic target in developing treatments for HCC [78, 79]. In conclusion, our study demonstrates the stability of TADs in the goose liver genomes induced by an HFD, except for the two genes *PRODH* and *GK*, which have the potential as a therapeutic target for HCC.

## Conclusions

In this study, geese exhibited significantly more significant alterations in liver index and TG content in response to an HFD than mice, without marked signs of inflammation. Our study elucidates how the dynamic three-dimensional chromatin architecture triggered by an HFD, which regulates genes that share homology with those in mice, plays crucial roles in lipid metabolism and is associated with liver diseases, NAFLD, or HCC. Investigating the dynamic three-dimensional chromatin structure in geese enhances our comprehension of the goose’s distinctive lipid metabolic and pathological adaptations.

### Supplementary Information

** Additional file 1: Fig. S1.** Data summary for the goose Hi-C data. The summary of total contacts (**A**), composition of alignable reads (**B**), unique reads (**C**) and valid contacts (**D**).

** Additional file 2: Fig. S2.** Gene expression in goose liver induced by high-fat diet (HFD). **A** Correlation matrix for goose liver mRNA profiles based on Pearson's correlation coefficient; **B** The transcriptome of goose HFD and normal liver with the typical NAFLD markers; **C** The gene enrichment analysis for the up-regulated DEGs in goose HFD liver tissues; **D** The gene enrichment analysis for the down-regulated DEGs in goose HFD liver tissues.

** Additional file 3: Fig. S3.** Data summary for the mouse Hi-C data. The summary of total contacts (**A**), composition of alignable reads (**B**) , unique reads (**C**) and valid contacts (**D**).

** Additional file 4: Fig. S4.** The Hi-C data access resolution for the HFD (**A**) and normal liver (**B**) groups in mouse.

** Additional file 5: Fig. S5.** The heatmaps display the correlation coefficients of HiCRep analysis for the mouse samples.

** Additional file 6: Fig. S6.** The distribution of topologically associating domains (TADs) was analyzed in both high-fat diet (HFD) and normal mouse liver tissues.

** Additional file 7: Table S1.** Statistical analysis of goose Hi-C data in goose liver.

**Additional file 8: Table S2.** Differentially expressed genes (DEGs) in high-fat diet (HFD) and normal goose liver tissues.

**Additional file 9: Table S3.** The functional analysis for the goose DEGs.

**Additional file 10: Table S4. **The goose gene list embedded in regions that experience the swith between Compartments A and B.

**Additional file 11: Table S5.** The functional analysis of the goose genes embedded in regions that experience the swith between Compartments A and B.

**Additional file 12: Table S6. **Genes within the reorganized topologically associating domains (TADs) of geese.

**Additional file 13: Table S7. **Genes in geese are regulated by the rewiring of promoter-enhancer interactions (PEIs).

**Additional file 14: Table S8. **Functional and pathway enrichment analyses for genes with significantly different RPS in the liver tissues of geese fed a high-fat diet (HFD).

**Additional file 15: Table S9.** Statistics of the mapping rate for mouse RNA-seq data.

**Additional file 16: Table S10. **Differentially expressed genes (DEGs) between mouse liver tissues from high-fat diet (HFD) and normal conditions.

**Additional file 17: Table S11. **Statistics of mouse Hi-C data.

**Additional file 18: Table S12.** List of mouse genes embedded in regions exhibiting a switch in Compartment A/B.

**Additional file 19: Table S13.** The genes located in the reorganization of mouse TADs.

**Additional file 20: Table S14. **Gene lists with significantly different RPS values between mouse liver tissues from high-fat diet (HFD) and normal conditions.

**Additional file 21: Table S15.** Functional analysis of differentially expressed genes (DEGs) in mice.

**Additional file 22: Table S16.** Functional analysis of mouse genes embedded in regions that undergo a switch in Compartment A/B.

**Additional file 23: Table S17. **Functional analysis of mouse genes with significantly different RPS values between mouse HFD and normal liver tissues.

**Additional file 24: Table S18. **Gene lists for the 6,988 one-to-one orthologous correspondence between geese and mouse.

**Additional file 25: Table S19. **Functional analysis of goose-specific genes regulated by the 3D genome architectures.

## Data Availability

The article’s multi-omics data concerning geese and mice can be accessed through the NCBI BioProject under the accession numbers PRJNA802613 and PRJNA739966. For detail, the geese RNA-seq data are available through the NCBI SRR17928987-17928990, SRR17928992-17928994, SRR17928996-17928,98; geese ChIP-seq data: SRR17930749-17930754; geese Hi-C data: SRR20278361-20278370. The mice RNA-seq data are available through the NCBI SRR14880475-SRR14880484; mice ChIP-seq data: SRR18026968-18026973; mice Hi-C data: SRR14880467-14880474, SRR14880485 and SRR14880486.

## Abbreviations

DEG: Differentially expressed gene
DI: Directionality index
FDR: False discovery rate
H3K27ac: H3K27 acetylation
HCC: Hepatocellular carcinoma
HFD: High-fat diet
Hi-C: High-throughput chromosome conformation capture
IS: Insulation score
NAFLD: Non-alcoholic fatty liver disease
PE: Poised enhancer
PEI: Promoter–enhancer interaction
RE: Regular enhancer
RNA-Seq: RNA sequencing data
RPS: Regulatory potential score
SE: Super-enhancer
TAD: Topologically associated domain
TCA: Tricarboxylic acid cycle
TG: Triglyceride
VNE: Von Neumann entropy
