# Supplementary figures and images for: Exploring the dynamic three-dimensional chromatin architecture and transcriptional landscape in goose liver tissues underlying metabolic adaptations induced by a high-fat diet
Source: J Anim Sci Biotechnol. 2024 May 2;15:60. doi: 10.1186/s40104-024-01016-5 (PMC11064361; doi:10.1186/s40104-024-01016-5)

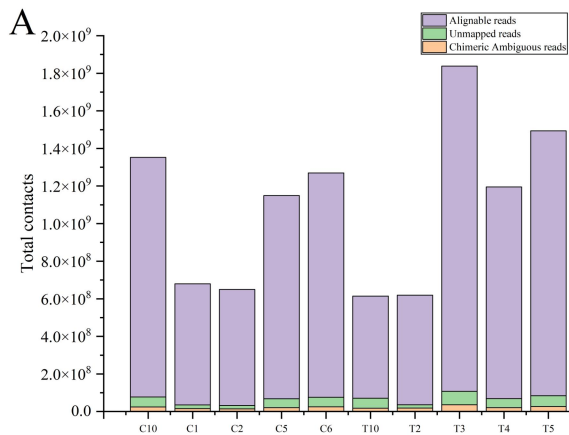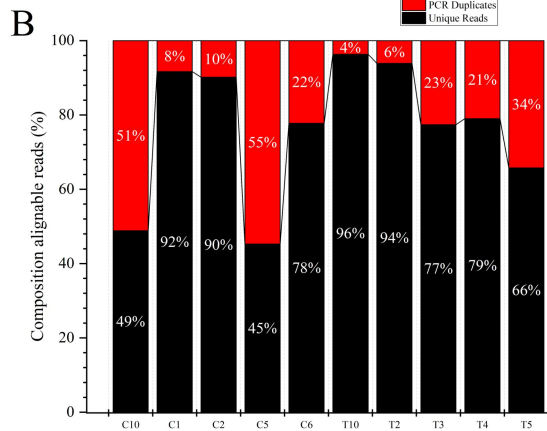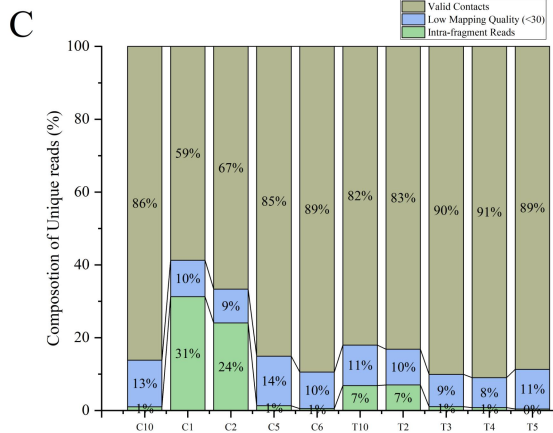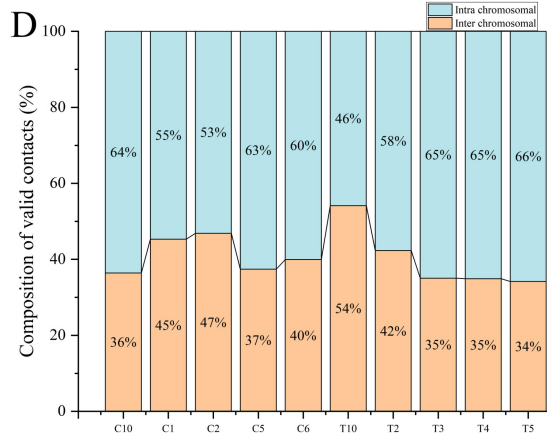

Supplement: Supplementary file 1 — Additional file 1: Fig. S1. Data summary for the goose Hi-C data. The summary of total contacts (A), composition of alignable reads (B), unique reads (C) and valid contacts (D). [file 40104_2024_1016_MOESM1_ESM.pdf]

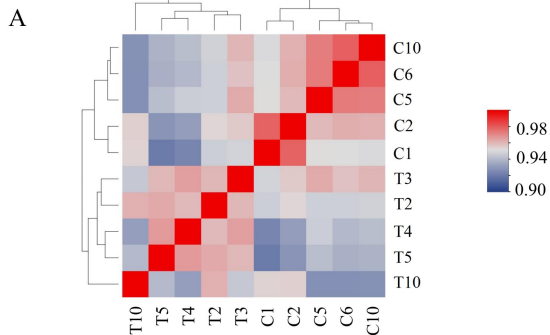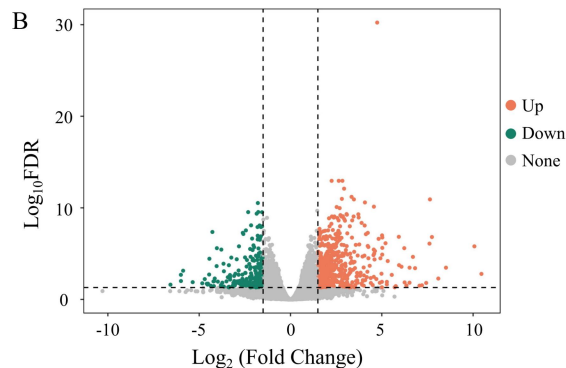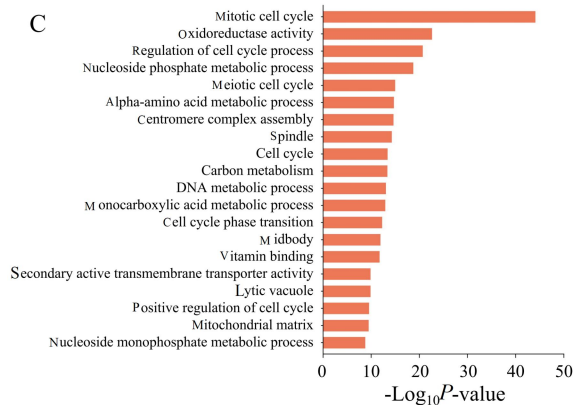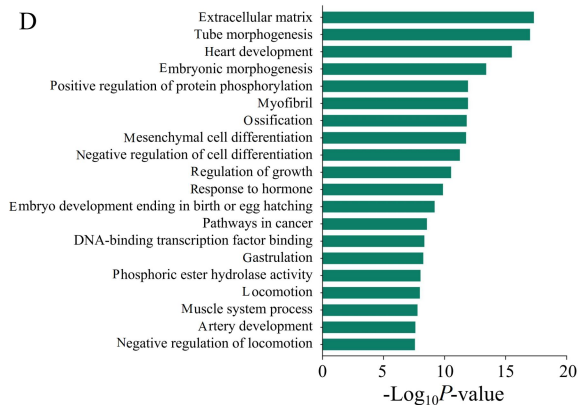

Supplement: Supplementary file 2 — Additional file 2: Fig. S2. Gene expression in goose liver induced by high-fat diet (HFD). A Correlation matrix for goose liver mRNA profiles based on Pearson's correlation coefficient; B The transcriptome of goose HFD and normal liver with the typical NAFLD markers; C The gene enrichment analysis for the up-regulated DEGs in goose HFD liver tissues; D The gene enrichment analysis for the down-regulated DEGs in goose HFD liver tissues. [file 40104_2024_1016_MOESM2_ESM.pdf]

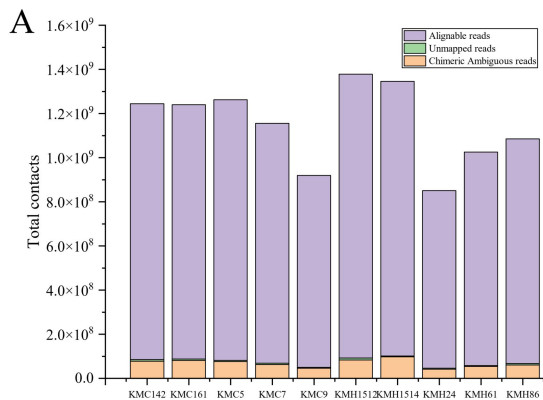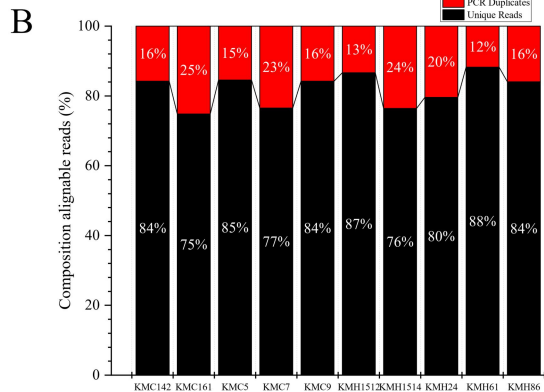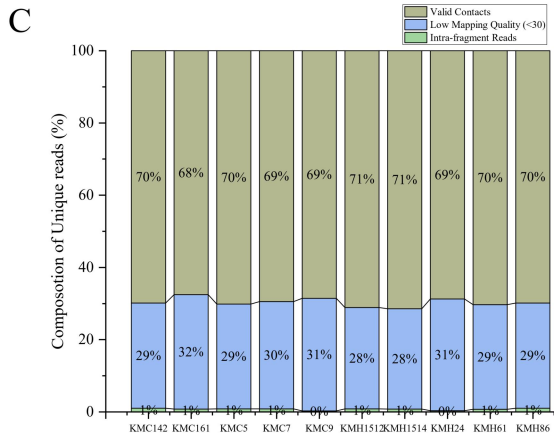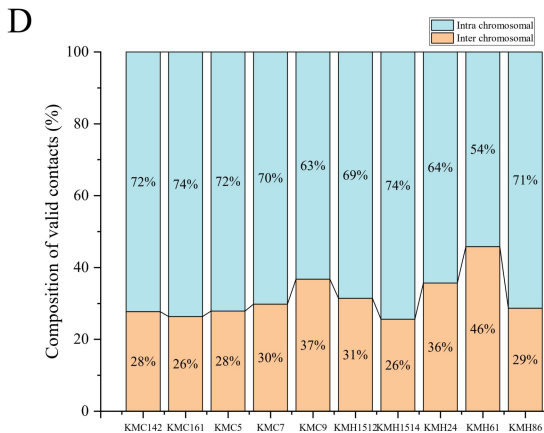

Supplement: Supplementary file 3 — Additional file 3: Fig. S3. Data summary for the mouse Hi-C data. The summary of total contacts (A), composition of alignable reads (B) , unique reads (C) and valid contacts (D). [file 40104_2024_1016_MOESM3_ESM.pdf]

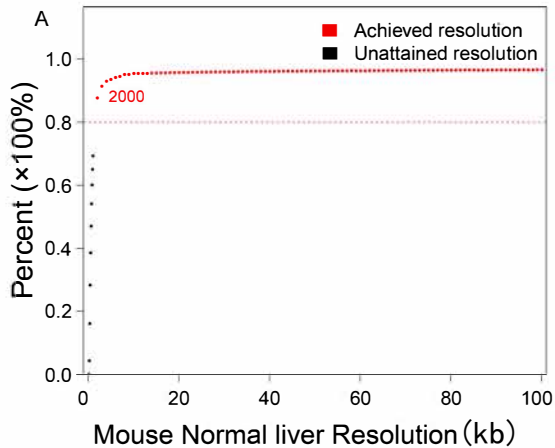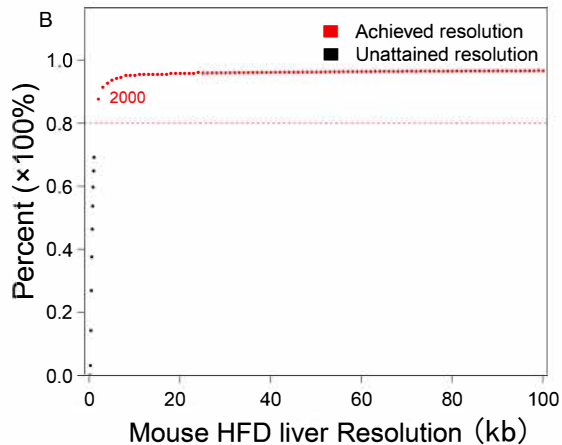

Supplement: Supplementary file 4 — Additional file 4: Fig. S4. The Hi-C data access resolution for the HFD (A) and normal liver (B) groups in mouse. [file 40104_2024_1016_MOESM4_ESM.pdf]

# mouse.hicrep-mcquitty

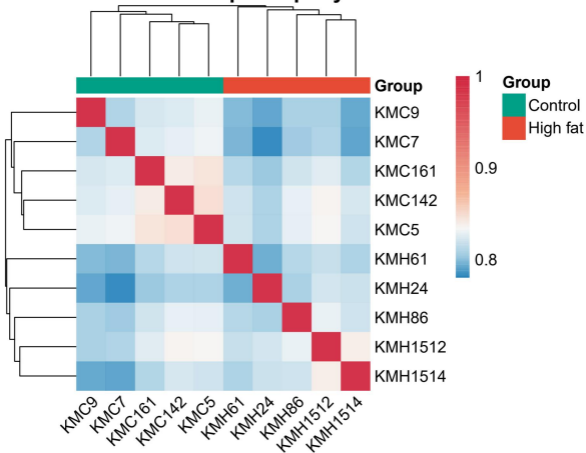

Supplement: Supplementary file 5 — Additional file 5: Fig. S5. The heatmaps display the correlation coefficients of HiCRep analysis for the mouse samples. [file 40104_2024_1016_MOESM5_ESM.pdf]

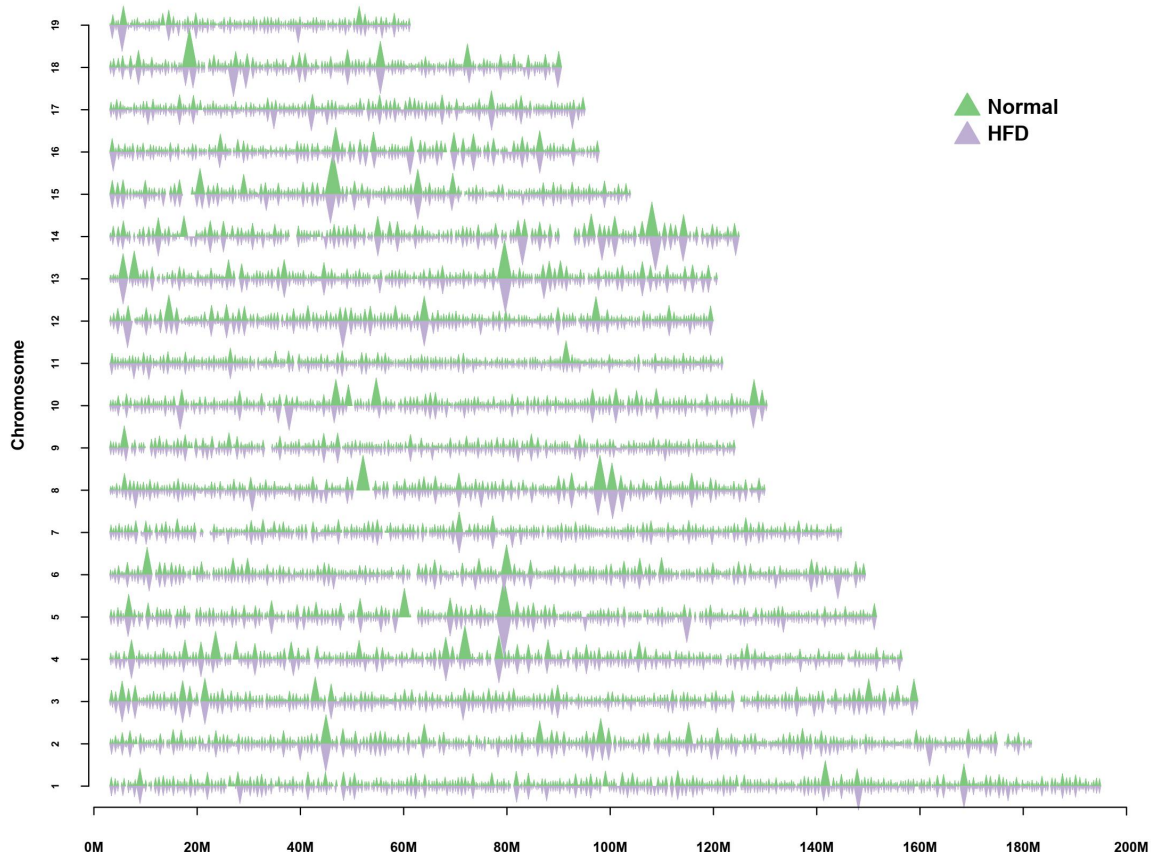

Supplement: Supplementary file 6 — Additional file 6: Fig. S6. The distribution of topologically associating domains (TADs) was analyzed in both high-fat diet (HFD) and normal mouse liver tissues. [file 40104_2024_1016_MOESM6_ESM.pdf]
